# Efficacy of Melatonin in Animal Models of Subarachnoid Hemorrhage: A Systematic Review and Stratified Meta-Analysis

**DOI:** 10.3389/fneur.2021.685731

**Published:** 2021-09-03

**Authors:** Xiangyu Hu, Yuwei Zhu, Fangfang Zhou, Cuiying Peng, Zhiping Hu, Chunli Chen

**Affiliations:** Department of Neurology, Second Xiangya Hospital, Central South University, Changsha, China

**Keywords:** subarachnoid hemorrhage, melatonin, systematic review, meta-analysis, animal models

## Abstract

**Background and Purpose:** Subarachnoid hemorrhage (SAH) is a severe disease characterized by sudden headache, loss of consciousness, or focal neurological deficits. Melatonin has been reported as a potential neuroprotective agent of SAH. It provides protective effects through the anti-inflammatory effects or the autophagy pathway. Our systematic review aims to evaluate the efficacy of melatonin administration on experimental SAH animals and offer support for the future clinical trial design of the melatonin treatment following SAH.

**Methods:** The following online databases were searched for experimentally controlled studies of the effect of melatonin on SAH models: PubMed, Web of Knowledge, Embase, and China National Knowledge Infrastructure (all until March 2021). The melatonin effect on the brain water content (BWC) and neurological score (NS) were compared between the treatment and control groups using the standardized mean difference (SMD).

**Results:** Our literature identified 160 possible articles, and most of them were excluded due to duplication (*n* = 69) and failure to meet the inclusion criteria (*n* = 56). After screening the remaining 35 articles in detail, we excluded half of them because of no relevant outcome measures (*n* = 16), no relevant interventions (*n* = 3), review articles (*n* = 1), duplicated publications (*n* = 1), and studies on humans or cells (*n* = 2). Finally, this systematic review contained 12 studies between 2008 and 2018. All studies were written in English except for one study in Chinese, and all of them showed the effect of melatonin on BWC and NS in SAH models.

**Conclusion:** Our research shows that melatonin can significantly improve the behavior and pathological results of SAH animal models. However, due to the small number of studies included in this meta-analysis, the experimental design and experimental method limitations should be considered when interpreting the results. Significant clinical and animal studies are still required to evaluate whether melatonin can be used in the adjuvant treatment of clinical SAH patients.

## Introduction

Although it only leads to 4.4% of all types of strokes, subarachnoid hemorrhage (SAH) is a severe disease with high mortality and morbidity (1). SAH is characterized by clinical features such as sudden headache, single or combined with vomiting, loss of consciousness, or focal neurological deficits (2). At present, the management and prevention of SAH are still challenging due to its intricate pathophysiological conditions (3). Rupture of intracranial aneurysms is a typical cause of SAH (4). Zero to 24 h after hemorrhage, early brain injury (EBI) is observed in focal (5), together with blood–brain barrier damage and vascular spasms (6). Further cellular changes include inflammation and autophagy. The released products mediate and persist inflammatory responses by danger-associated molecular patterns (DAMPs) (7). Activation of the MAPK (mitogen-activated protein kinase) and Keap1-Nrf2-ARE [(Kelch-like ECH-Associating protein 1) nuclear factor erythroid 2 related factor 2-antioxidant response element] pathways may take over part of the inflammatory damage mechanism (8, 9). The EBI following SAH can also be induced by the autophagy mechanism (10), with the activation of mitochondria and the downstream pathway (11).

Recently, melatonin (N-acetyl-5-methoxytryptamine) has been reported as a potential neuroprotective agent of SAH. Melatonin, which derives from tryptophan (12), was demonstrated to counterwork oxidative stress and assist in scavenging free radicals (13). In 2010, researchers raised concerns about the anti-inflammatory effects of melatonin, specifically those comprising the reduction of the pathological changes in the tissues, attenuation of the development of O_2_-induced hyperalgesia and blockage of cyclooxygenase-2 (COX-2), and inducible nitric oxide synthase (iNOS) induction (14). Additionally, melatonin provides protective effects through the autophagy pathway in the Senescence Accelerated Mouse-Prone 8 (SAMP8) mice (15). It seems that melatonin plays an effective neuroprotective role in SAH management (16, 17). However, this argument is inconsistent (18). The current study aims to evaluate and validate the efficacy of melatonin administration on experimental SAH animals. The related factors of research design that could shape the results will also be analyzed. Further, this preclinical study may offer support for the future clinical trial design of the melatonin treatment following SAH.

## Methods

### Data Sources, Search Strategy, and Selection Criteria

The following online databases were searched for experimentally controlled studies of the effect of melatonin on SAH models: PubMed, Web of Knowledge, Embase, and China National Knowledge Infrastructure (all until March 2021). The following search terms were used: (subarachnoid h(a)emorrhage OR SAH OR aneurysm) AND (melatonin OR N-acetyl-5-methoxytryptamine OR melatonergic agent) NOT human NOT patient. The reference lists of the included studies were also searched. Studies that met the following selection criteria were included: (a) experimental SAH was induced; (b) melatonin was administered before or after the induction of SAH; (c) control animals were used; (d) no co-treatments were performed; (e) therapeutic effects of melatonin were assessed by brain water content or neurobehavioral outcome.

### Data Extraction

Two authors (XiangYu Hu and YuWei Zhu) independently extracted data from studies according to the selection criteria based on animal species, gender, number, intervention (dose and time of melatonin treatment), anesthetic technique, SAH induction method, measured outcomes, assessment time, and methodological quality score. Disagreements were addressed by a discussion with a third author (ChunLi Chen). When the included studies used multiple groups to assess dose–response relationships, we extracted data from each group individually. Graphically presented data were measured by the GetData Graph Digitizer software (version 2.26). The animal quantity, mean score, or content and standard deviation (SD), or standard error of the mean (SEM) in the melatonin and control groups were collected. When neurological tests were managed at different time points, only the final point was included. When it was unclear whether SD or SEM was the measure of variance, data was extracted as the SD due to it being a more accurate estimate, according to the present meta-analysis.

### Quality Assessment

The methodological quality was evaluated using the collaborative approach to meta-analysis and review of Animal Data from Experimental Studies (CAMARADES) 10-item checklist with minor modifications (19). One point was given for each of the following criteria: peer-reviewed publication, control of temperature, random allocation to treatment or control, blinded induction of hemorrhage, blinded assessment of outcome, use of anesthetic without marked intrinsic neuroprotective activity, appropriate animal model (aged, diabetic or hypertensive), sample size calculation, compliance with animal welfare regulations, and statement of potential conflict of interests.

### Data Analysis

A statistical software package (Stata, version 12.0; StataCorp LP, College Station, TX, USA) was used to perform data analyses. The melatonin effect on the brain water content (BWC) and neurological score (NS) were compared between the treatment and control groups using the standardized mean difference (the difference in the effect of melatonin between the treatment and control groups was divided by the total SD). This allowed for comparisons to be made when various measure methods or animal species were being used. The DerSimonian-Laird random-effects model was adopted to comprehensively estimate the effect size (20). We conducted a stratified meta-analysis to clarify the impact of drug dose, time of administration, overall study quality score, method of SAH induction, animal species, and type of anesthetic used. Funnel plotting was used to detect publication bias, the asymmetry of which was evaluated using Egger's test and the trim-and-fill method (21). Statistical significance was set at *P* < 0.05 and the 95% confidence intervals (CIs) were calculated.

## Results

### Study Characteristics

**Figure 1:**
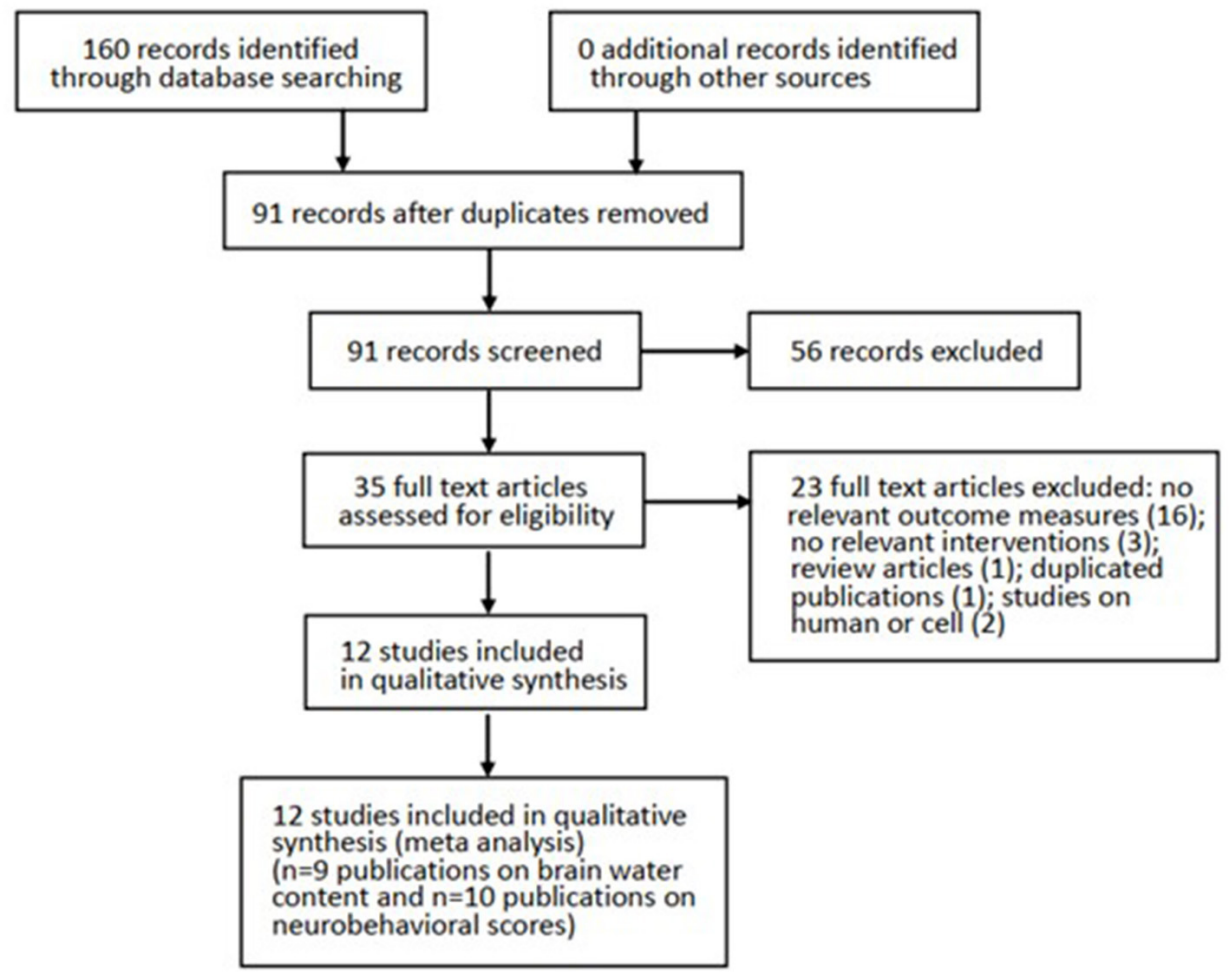
Progression from literature search to meta-analysis. The number of exclusions from the initial literature search is shown.

Our literature identified 160 possible articles, although most of them were excluded due to duplication (*n* = 69) and failure to meet the inclusion criteria (*n* = 56). After screening the remaining 35 articles in detail, we excluded half of them because of no relevant outcome measures (*n* = 16), no relevant interventions (*n* = 3), review articles (*n* = 1), duplicated publications (*n* = 1), and studies on humans or cells (*n* = 2). Finally, this systematic review contained 12 studies between 2008 and 2018. All studies were written in English except for one study in Chinese, and all of them showed the effect of melatonin on BWC and/or NS in SAH models (Figure 1).

We extracted data from 12 studies that describe BWC in 10 studies (26 comparisons) and NS in 12 studies (13 comparisons); the overall study characteristics are shown in Table 1. Among them, nine studies used SD rats, two studies used C57 mice, and one study used Wistar rats. Most of the studies involved male and adult models; only one study did not report the gender of animals. Regarding anesthesia, seven studies used pentobarbital, four studies used an anesthetic containing ketamine, and one study was done with isoflurane. All drugs were administered by intraperitoneal injection. Further, nine studies used endovascular perforation to induce the SAH model; the remaining three studies induced the model with autogenous blood injection. In most studies, the common initial drug dosage was 150 mg/kg, but one study treated the rats with an initial drug dosage of 5 mg/kg. Most studies performed the treatment of melatonin in 2 h after the induction of SAH while some included repeated injections. The melatonin treatment course was up to 72 h. Assessments were performed 24–56 h after the induction of SAH.

**Table 1:** Design characteristics of included studies.

**References**	**Animal,** ** sex**	**Age**	**Anesthetic**	**Route**	**Method of SAH**	**Initial dose**	**Total dose**	**Time to treatment**	**Tre (*n*)/Con (*n*)**	**Assessment time**	**Outcome measure (direction)**
Ayer et al. (22)	SD rats, Male	Adult	Ketamine and Xylazine	i.p.	Endovascular perforation	150 mg/kg	150 mg/kg	2 h post SAH	6,7	24 h	BWC (lower is better);
Ersahin et al. (23)	Wistar rats, Male	Adult	ketamine and chlorpromazine	i.p.	Autogenous blood	10 mg/kg	30 mg/kg	Immediately post SAH (every 24 h for 2 days)	6,6 6,6	48 h	BWC (lower is better); NS (lower is better)
Wang et al. (24)	SD rats, Male	Adult	ketamine and xylazine	i.p.	Autogenous blood	150 mg/kg	300 mg/kg	2 and 24 h post SAH	6,6	48 h	BWC (lower is better);
Cai et al. (25)	SD rats, NR	Adult	Ketamine and xylazine	i.p.	Autogenous blood	15 mg/kg	60 mg/kg	5 min, 24, 48, and 72 h post SAH	8,8	72 h	NS (higher is better)
Chen et al. (26)	SD rats, Male	Adult	pentobarbital	i.p.	Endovascular perforation	150 mg/kg	150 mg/kg	2 h post SAH	5,5 25,24	24 h	BWC (lower is better); NS (higher is better)
Chen et al. (17)	SD rats, Male	Adult	pentobarbital	i.p.	Endovascular perforation	150 mg/kg	150 mg/kg	2 h post SAH	6,6 24,24	24 h	BWC (lower is better); NS (higher is better)
Chen et al. (27)	SD rats, Male	Adult	pentobarbital	i.p.	Endovascular perforation	150 mg/kg	150 mg/kg	2 h post SAH	20,17	24 h	NS (higher is better)
Dong et al. (28)	C57 mice, Male	Adult	isoflurane	i.p.	Endovascular perforation	150 mg/kg	150 mg/kg	2 h post SAH	6,6 39,37	24 h	BWC (lower is better) NS (higher is better)
Zhao et al. (29)	C57 mice, Male	Adult	pentobarbital	i.p.	Endovascular perforation	150 mg/kg	300 mg/kg	2 and 12 h post SAH	6.6	24 h	BWC (lower is better); NS (higher is better)
Yu (30)	SD rats, Male	Adult	pentobarbital	i.p.	Endovascular perforation	150 mg/kg	450 mg/kg	2, 24, and 48 h post SAH	26,30	72 h	NS (higher is better)
Yu (30)	SD rats, Male	Adult	pentobarbital	i.p.	Endovascular perforation	150 mg/kg	150 mg/kg	2 h post SAH	6,6 24,24	24 h	BWC (lower is better); NS (higher is better)
Cao et al. (31)	SD rats, Male	Adult	pentobarbital	i.p.	Endovascular perforation	150 mg/kg	150 mg/kg	2 h post SAH	6,6 24,24	24 h	BWC (lower is better); NS (higher is better)
Shi et al. (32)	SD rats, Male	Adult	pentobarbital	i.p.	Endovascular perforation	5 mg/kg;10 mg/kg	5 mg/kg; 10 mg/kg	2 h post SAH	6,6 18,18	24 h	BWC (lower is better); NS (higher is better)
Shi et al. (32)	SD rats, Male	Adult	pentobarbital	i.p.	Endovascular perforation	10 mg/kg	10 mg/kg	2 h post SAH	12,12	24 h	NS (higher is better)

*SAH, subarachnoid hemorrhage; Tre, treated; Con, control; i.p., intraperitoneal; BWC, brain water content; NS, Neurobehavioral score; NR, not reported*.

### Study Quality

Our systematic review included 12 studies in which the quality scores varied from three to seven. Among them, one study was assessed as a poor methodological quality study. Further, all the scores of others were higher than or equal to 4; their mean value (interquartile range) was 5.92 (1.5). All studies were peer-reviewed publications. Nine (75%) studies were completed with the control of temperature, the same number of studies allocated animals to treatment or control randomly, and 10 (83%) studies used a blind assessment to evaluate outcomes.

For the assessment of BWC, the median number (interquartile range) of animals in the treatment and control groups was 6 (0). For the assessment of NS, the median numbers (interquartile range) of animals in the treatment and control groups were 24 (15.5) and 21 (14), respectively. The details of the quality index are presented in Table 2.

**Table 2:** Methodological quality of 12 studies included in the meta-analysis.

**Study**	**(1)**	**(2)**	**(3)**	**(4)**	**(5)**	**(6)**	**(7)**	**(8)**	**(9)**	**(10)**	**Total**
Ayer et al. (22)	√	√								√	3
Ersahin et al. (23)	√	√			√				√	√	5
Wang et al. (24)	√	√			√				√	√	5
Cai et al. (25)	√	√	√		√				√	√	6
Chen et al. (26)	√	√	√		√	√			√	√	7
Chen et al. (17)	√	√	√		√	√			√	√	7
Chen et al. (27)	√		√		√	√			√	√	6
Dong et al. (28)	√	√	√		√	√			√	√	7
Zhao et al. (29)	√	√	√		√	√			√	√	7
Yu (30)	√	√	√			√			√	√	6
Cao et al. (31)	√		√		√	√			√	√	6
Shi et al. (32)	√	√	√		√	√			√	√	7

*(1) peer reviewed publication; (2) control of temperature; (3) random allocation to treatment or control; (4) blinded induction of hemorrhage; (5) blinded assessment of outcome; (6) use of anesthetic without marked intrinsic neuroprotective activity; (7) animal model (aged, diabetic, or hypertensive); (8) sample size calculation; (9) compliance with animal welfare regulations; and (10) statement of potential conflict of interests*.

### Global Estimates of Efficacy

**Figure 2:**
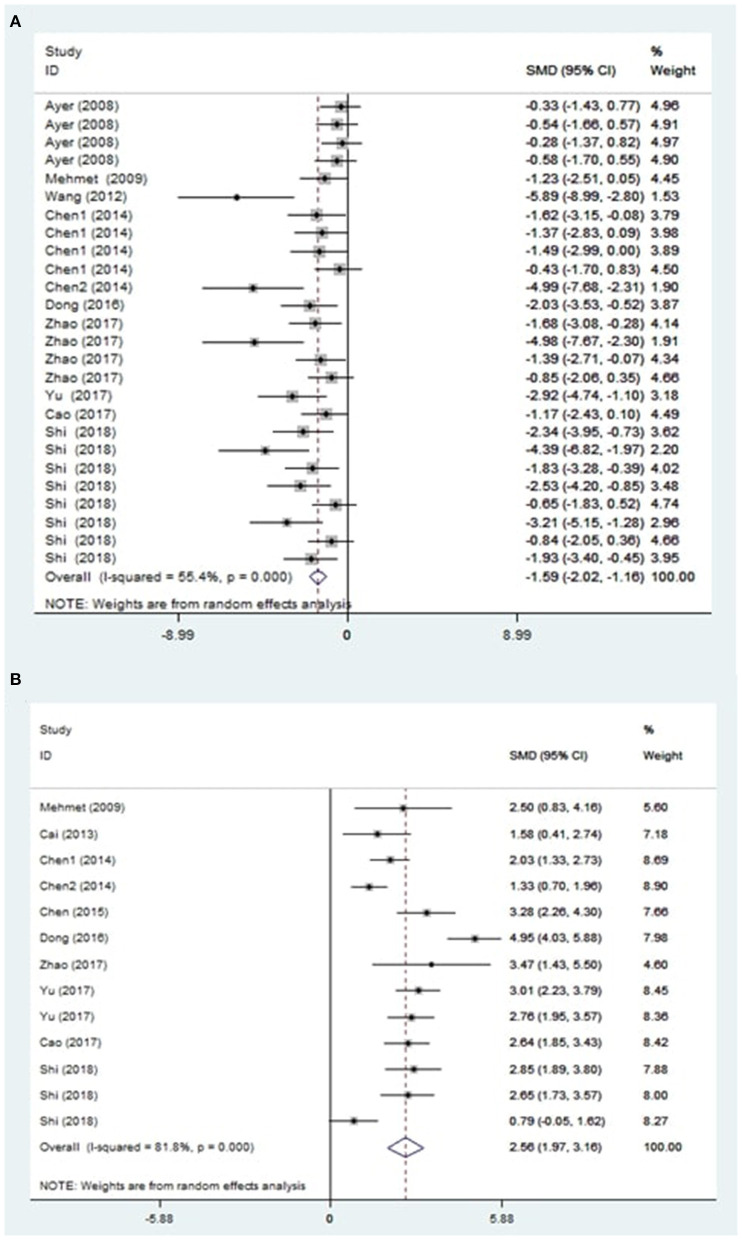
Effect sizes of included comparisons. A forest plot shows mean effect size and 95% CI for **(A)** brain water content and **(B)** neurobehavioral outcomes.

The treatment of melatonin showed a significant reduction in the BWC by an SMD of −1.59 (95% CI: −2.02, −1.16; *p* < 0.0001, 10 studies, 26 comparisons, Figure 2A), with a statistically significant heterogeneity (Q = 56.02, *I*^2^ = 55.4%, df = 25, *p* < 0.001).

On the other hand, the global estimate of the efficacy of melatonin in improving the NS outcomes was 2.56 (95% CI: 1.97, 3.16; *p* < 0.0001, 10 studies, 13 comparisons, Figure 2B). The heterogeneity of NS outcomes among comparisons was also statistically significant (Q = 66.08, *I*^2^ = 81.8%, df = 12, *p* < 0.001). Therefore, further subgroup analysis was performed.

### Sensitivity Analysis

**Figure 3:**
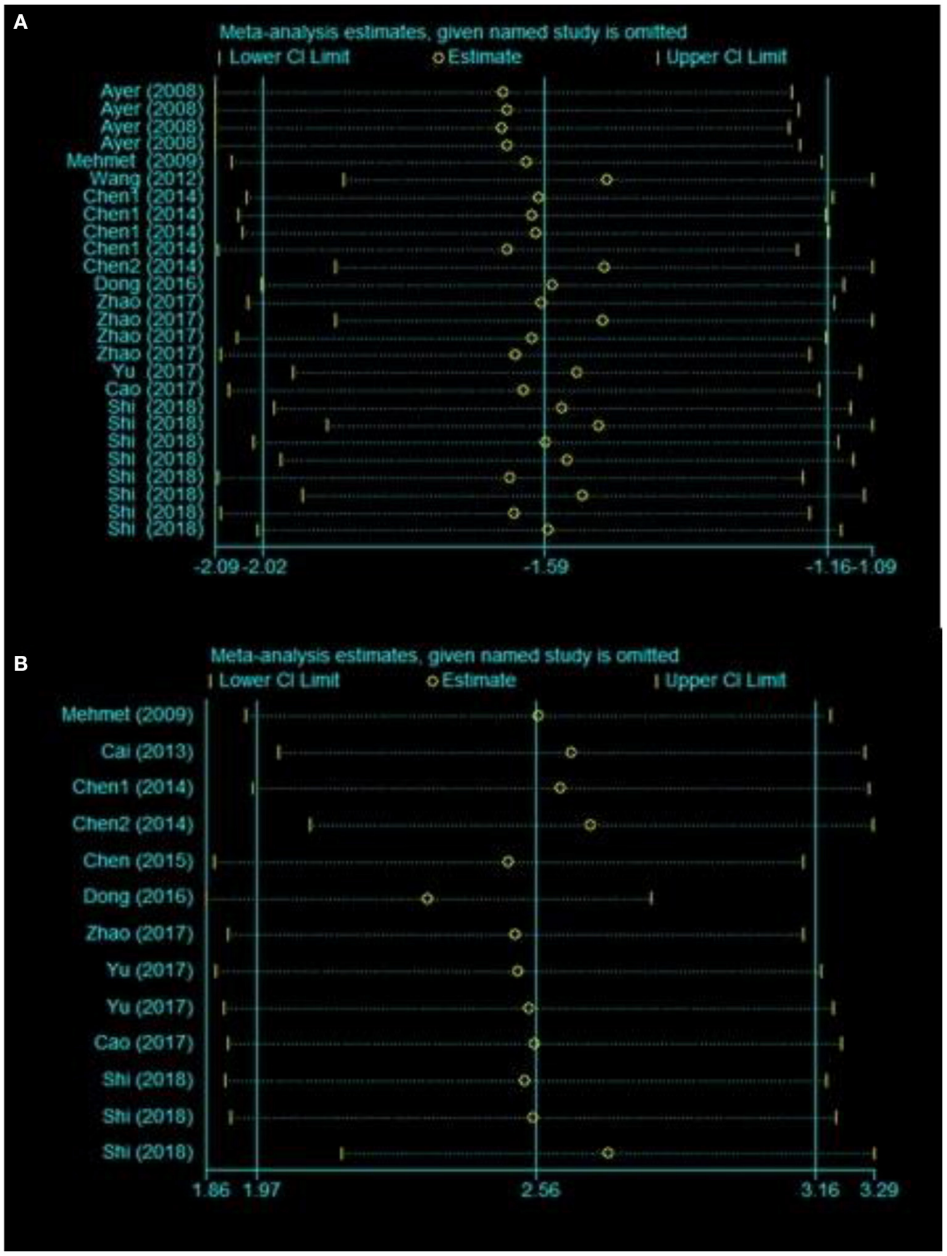
Sensitivity analysis for the included comparisons. Figures shows mean effect size and 95% CI for **(A)** brain water content and **(B)** neurobehavioral outcomes.

To evaluate the stability of the results, we performed a sensitivity analysis through the sequential omission of each study. For the pooled SMD, neither brain water content nor neurobehavioral outcomes were significantly affected by any study (Figures 3A,B).

### Publication Bias

**Figure 4:**
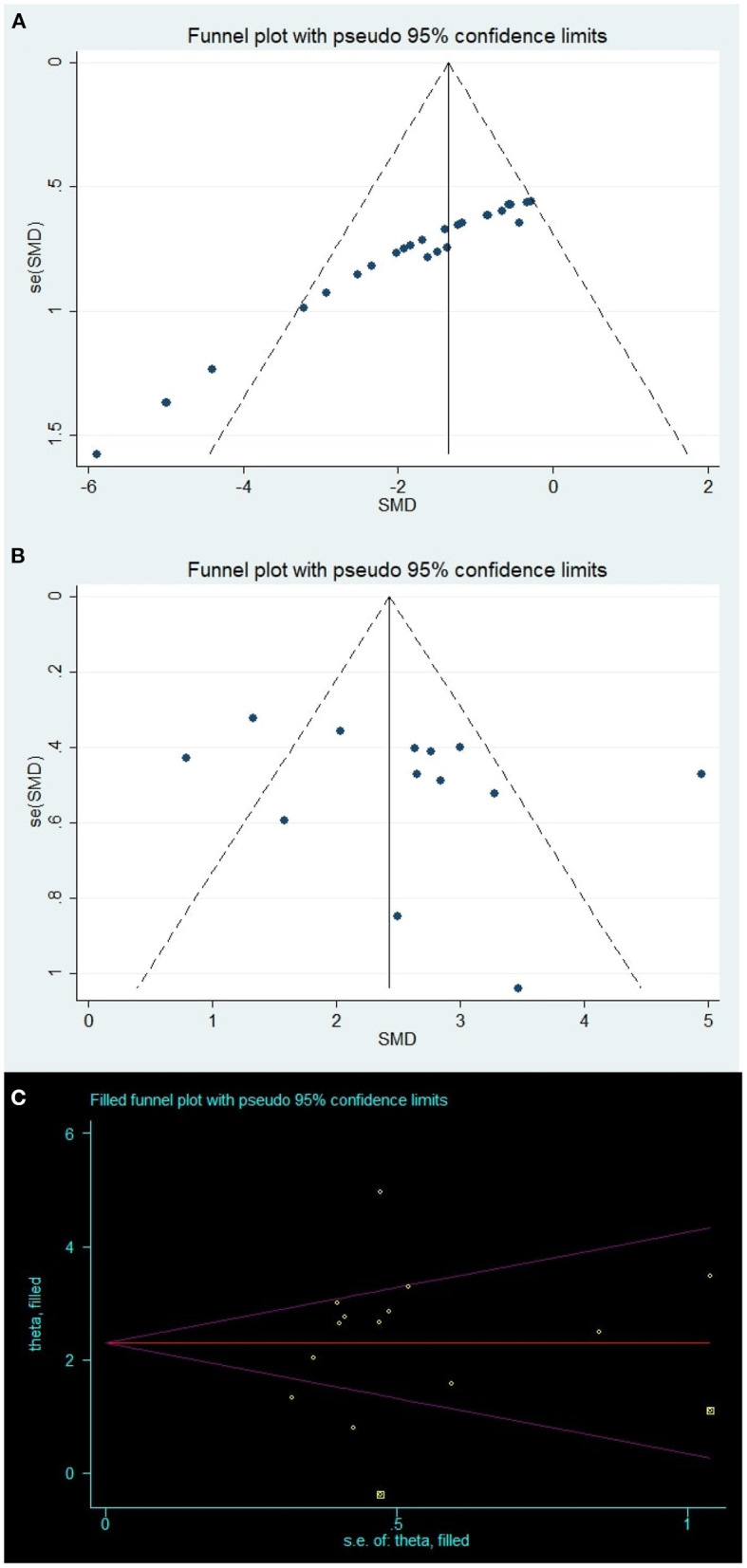
Publication bias. Funnel plots for **(A)** brain water content and **(B)** neurobehavioral outcomes; Yellow points in **(C)** represent theoretically missing comparisons identified using the trim-and-fill method.

Visual inspection of the funnel plot suggested conspicuous publication bias for the brain water content (Figure 4A), and the results of the Egger test suggested the same comment (*p* < 0.001). The trim-and-fill analysis was used to estimate the missing studies (SMD: −1.59, 95% CI: −2.02, −1.16; *p* < 0.001), indicating no “missing” studies.

For the neurobehavioral outcomes, the funnel plot was approximately symmetrical (Figure 4B) and the Egger test indicated no significant publication bias (p = 0.305). However, two theoretically missing comparisons of neurobehavioral outcomes were predicted (Figure 4C).

### Subgroup analysis

We performed a subgroup analysis of brain water content and neurobehavioral score; the details of the data are shown in Tables 3, 4.

**Table 3:** Stratified meta-analysis of heterogeneity on brain water content.

**Subgroup analysis**	**No. of studies**	**SMD (95%CI)**	**Heterogeneity test**		
			**Q**	***I*^**2**^**	***p***
**1.1 Study quality**
High	22	−1.86 (−2.33, −1.40)	41.65	50%	0.005
Low	4	−0.43 (−0.98, 0.13)	0.21	0%	0.98
**1.2 Method to induce SAH**
Endovascular perforation	24	−1.52 (−1.94, −1.09)	47.63	52%	0.002
Autogenous blood	2	−3.34 (−7.89, 1.21)	7.46	87%	0.006
**1.3 Anesthetic drugs**
Pentobarbital	19	−1.79 (−2.28, −1.31)	33.74	47%	0.01
Ketamine	6	−0.89 (−1.72, −0.05)	12.63	60%	0.03
Isoflurane	1	−2.03 (−3.53, −0.52)			
**1.4 Dosage of melatonin**
5 mg/kg	4	−1.28 (−2.04, −0.53)	3.83	22%	0.28
10 mg/kg	5	−2.38 (−3.35, −1.41)	6.68	40%	0.15
150 mg/kg	17	−1.46 (−2.01, −0.91)	39.11	59%	0.001
**1.5 Dose administration**
Single dose	20	−1.48 (−1.94, −1.01)	39.55	52%	0.004
Repeat dose	6	−2.11 (−3.27, −0.95)	15.41	68%	0.009

**Table 4:** Stratified meta-analysis of heterogeneity on neurobehavioral score.

**Subgroup analysis**	**No. of studies**	**SMD (95%CI)**	**Heterogeneity test**		
			**Q**	***I*^**2**^**	***p***
**1.1 Method to induce SAH**
endovascular perforation	11	2.65 (1.99, 3.31)	63.94	84%	<0.001
autogenous blood	2	1.88 (0.93, 2.84)	0.78	0%	0.38
**1.2 Anesthetic drugs**
pentobarbital	10	2.39 (1.86, 2.92)	33.95	73%	<0.001
ketamine	2	1.88 (0.93, 2.84)	0.78	0%	0.38
isoflurane	1	4.95 (4.03, 5.88)			
**1.3 Dosage of melatonin**
5 mg/kg	1	2.85 (1.89, 3.80)			
10 mg/kg	3	1.91 (0.55, 3.28)	9.56	79%	0.008
15 mg/kg	1	1.58 (0.41, 2.74)			
150 mg/kg	8	2.87 (2.09, 3.65)	46.63	85%	<0.001
**1.4 Dose administration**
Single dose	9	2.56 (1.80, 3.32)	60.84	87%	<0.001
Repeat dose	4	2.57 (1.77, 3.37)	4.69	36%	0.20

For brain water content, we stratified the data by study quality; the high-quality studies showed a higher effect size (SMD: −1.86; 95% CI: −2.33, −1.40; *p* < 0.001, Supplementary Figure 1) and the results of the low-quality studies were not statistically significant.

Different SAH induction methods were used in studies, and our subgroup analysis showed that the results of the autogenous blood model (SMD: −3.34; 95% CI: −7.89, 1.21; *p* = 0.15, Supplementary Figure 2) were more effective than those of the endovascular perforation model with no statistical significance.

Concerning the anesthesia drug, the studies that used isoflurane exhibited a higher effect size than others (SMD: −2.03; 95% CI: −3.53, −0.52; *p* < 0.001, Supplementary Figure 3). However, the small sample size makes the assessments less reliable.

The dosage of melatonin ranged from 5 to 150 mg/kg, and the greatest effect was exerted at the dosage of 10 mg/kg (SMD: −2.38; 95% CI: −3.35, −1.41; *p* < 0.001, Supplementary Figure 4). Further, we found the repeated melatonin dosage showed a greater effect size than a single dosage (SMD: −2.11; 95% CI: −3.27, −0.95; *p* < 0.001, Supplementary Figure 5).

For the neurobehavioral score, our subgroup analysis showed that the endovascular perforation model exhibited a higher effect size than the autogenous blood model (SMD: 2.65; 95% CI: 1.99, 3.31; *p* < 0.001, Supplementary Figure 6).

The studies that used isoflurane showed a higher effect size than other anesthetics (SMD: 4.95; 95% CI: 4.03, 5.88; *p* < 0.001, Supplementary Figure 7), which was consistent with the results of the brain water content.

The dosage of melatonin ranged from 5 to 150 mg/kg, and in a result different from that of brain water content, the studies using a dosage of 150 mg/kg exhibited the highest effect size (SMD: 2.39; 95% CI: 1.86, 2.92; *p* < 0.001, Supplementary Figure 8).

Besides, we stratified the data by dose administration, and there was no significant effect size difference in neuroprotective effects for both the doses administered (Supplementary Figure 9).

## Discussion

### Summary of Evidence

Although there have been related systematic reviews on melatonin in experimental cerebral ischemia (33) and traumatic brain injury (34), as far as we know, this is the first systematic review and meta-analysis of melatonin in an animal model with SAH. The outcomes of our systematic review indicate that melatonin shows significant neuroprotective effects in reducing brain water content [SMD: −1.59 (95% CI: −2.02, −1.16)] and improving neurobehavioral outcomes [SMD: 2.56 (95% CI: 1.97, 3.16)]. These findings signify that melatonin can be used as a potential treatment for clinical SAH. However, due to the limited number of studies and poor literature quality, more studies are needed to prove the neuroprotective effect of melatonin in experimental SAH.

### Interpretation of Subgroup Analysis

As a basic type of stroke, SAH has received more and more attention because of its high morbidity and mortality. The early-stage-damage of SAH is related mainly to the impaired brain autoregulation. The subsequent cerebral vasospasm/delayed cerebral ischemia significantly affects patients' prognosis (35, 36). As a non-toxic antioxidant, melatonin has attracted much attention because of good tolerance and high penetration level through the blood-brain barrier. It is generally believed that melatonin can exert its neuroprotective effects on SAH in many aspects. On the one hand, melatonin can enhance early-stage brain autoregulation through inhibiting sympathetic nerve activity, mediating myogenic response, alleviating hypoxemia, and regulating metabolism. On the other hand, melatonin plays an antioxidant role to protect endothelial cell function, increase nitric oxide availability, and relieve vasospasm/delayed cerebral ischemia (37). In this study, melatonin presented significant effects on improving neurological score and ameliorating cerebral edema. Nevertheless, due to obvious heterogeneity of the result, the next step of subgroup analysis was performed. The results show that methodological differences including modeling methods, anesthetic drugs, the dosage of melatonin, and dose administration have contributed to the heterogeneity of the results.

For brain water content, the results of the subgroup analysis showed that melatonin has a higher efficacy in the high study quality group. Previous studies have found that research quality has a significant impact on the outcome; it is easier to exaggerate the effect size in low-quality studies (38, 39). However, some studies believe that the research quality has no significant influence on the effect size (40). In our meta-analysis, although the high-quality studies show better results compared with the low-quality studies, more research is needed to get a positive result, considering the limited number of low-quality studies.

There is no clear relationship between the modeling methods and effect size. For brain water content, the autologous blood injection model achieved a better result with no statistical significance, but the opposite results were achieved in the neurobehavioral score group, wherein the endovascular perforation model was more effective. At present, no animal model can simulate the physiological conditions of human subarachnoid hemorrhage. For the autologous blood injection model, the volume of blood and the injection site varied in different studies. For the endovascular perforation model, the sutures used for puncture were different. Besides, the types of rats and animal weights included in our research were also not the same. It can be considered that the lack of a unified SAH animal model is the main reason for the differentiated results (41, 42).

Our results showed that isoflurane achieved the best effect in both the brain water content and neurobehavioral score groups, but the results are less reliable considering the small sample size. In fact, the neuroprotective effects of phenobarbital, isoflurane, and ketamine anesthetics have been widely reported (43–45), and the specific mechanism may be related to the anesthetic's inhibition of GABA-a inhibitory and NMDA excitatory receptors (46, 47). Although the effect of anesthetics on the pathophysiological process of subarachnoid hemorrhage is still unclear, the potential neuroprotective effect cannot be ignored when the results are interpreted.

The dosage of melatonin ranged from 5 to 150 mg/kg. In the brain water content group, 10 mg/kg achieved the greatest effect size, while in the neurobehavioral score group, 150 mg/kg had the best effect size. It is difficult to conclude at what dosage the drug achieves the best effect due to the wide range of drugs. Furthermore, the results showed that the repeated doses had greater efficacy than the single doses, which indicated a short duration of action after single-dose treatment. This may be further related to the fact that the metabolic half-life of melatonin in rats by oral or intravenous injection was <20 min (48).

### Advantages and Limitation

Our study made a lot of effort to obtain relatively objective results. First, this meta-analysis collected the most comprehensive reports in this field possible, representing the most complete analysis of the use of melatonin in SAH animal models. Second, two experienced researchers independently evaluated and extracted all the data studies included to reduce potential publication bias. Finally, our results showed that both the behavior and pathological results of SAH animal models treated with melatonin showed significant improvement, indicating melatonin can probably be used as a new treatment strategy in the treatment of clinical SAH patients.

Although this study achieved positive results, the possible shortcomings should not be ignored. First, our research only includes the available data; some negative results are less likely to have been published. The cut-and-fill method shows two theoretically missing studies on the neurobehavioral score group. Therefore, this meta-analysis may have exaggerated the effect size. Second, the studies we included were highly heterogeneous with an *I*^2^ of 55.4 and 81.8%, which may be related to animal species, study quality, modeling methods, anesthetics, drug dosage, and dose administration; therefore, a further subgroup analysis was carried out. However, due to the limited sample size of each subgroup and insufficient statistical power, the differences between some subgroups were not obvious. Third, our study still lacks the findings of the effect of melatonin in SAH rat models with specific types of diseases (such as diabetes and hypertension). The clinical patients are more likely to present with different types of underlying diseases, so there is still a lot of work to be done in clinical translation.

## Conclusion

Our research shows that melatonin can significantly improve the behavior and pathological results of SAH animal models. However, due to the small number of studies included in this meta-analysis, the experimental design and experimental method limitations should be considered when interpreting the results. Significant clinical and animal studies are still required to evaluate whether melatonin can be used in the adjuvant treatment of clinical SAH patients.

## Data Availability Statement

The original contributions presented in the study are included in the article/Supplementary Material, further inquiries can be directed to the corresponding author/s.

## Author Contributions

ZH: study concept and design. XH and YZ: acquisition of data. CC, CP, and FZ: analysis and interpretation of data. XH and YZ: drafting of the manuscript. CC and ZH: revision of manuscript. CC: supervision of work. All authors read and approved the final manuscript.

## Conflict of Interest

The authors declare that the research was conducted in the absence of any commercial or financial relationships that could be construed as a potential conflict of interest.

## Publisher's Note

All claims expressed in this article are solely those of the authors and do not necessarily represent those of their affiliated organizations, or those of the publisher, the editors and the reviewers. Any product that may be evaluated in this article, or claim that may be made by its manufacturer, is not guaranteed or endorsed by the publisher.

## References

[1] Wang W Jiang B Sun H Ru X Sun D Wang L. Prevalence, incidence, and mortality of stroke in china: results from a nationwide population-based survey of 480 687 adults. Circulation. (2017) 135:759–71. 10.1161/CIRCULATIONAHA.116.025250 28052979

[2] van Gijn J Kerr RS Rinkel GJ. Subarachnoid haemorrhage. Lancet. (2007) 369:306–18. 10.1016/S0140-6736(07)60153-6 17258671

[3] Gareev I Beylerli O Aliev G Pavlov V Izmailov A Zhang Y. The role of long non-coding RNAs in intracranial aneurysms and subarachnoid hemorrhage. Life. (2020) 10:155. 10.3390/life10090155 32825276 PMC7555693

[4] Molyneux AJ Kerr RS Yu LM Clarke M Sneade M Yarnold JA. International subarachnoid aneurysm trial (ISAT) of neurosurgical clipping versus endovascular coiling in 2143 patients with ruptured intracranial aneurysms: a randomised comparison of effects on survival, dependency, seizures, rebleeding, subgroups, and aneurysm occlusion. Lancet. (2005) 366:809–17. 10.1016/S0140-6736(05)67214-5 16139655

[5] Park S Yamaguchi M Zhou C Calvert JW Tang J Zhang JH. Neurovascular protection reduces early brain injury after subarachnoid hemorrhage. Stroke. (2004) 35:2412–7. 10.1161/01.STR.0000141162.29864.e9 15322302

[6] Provencio JJ Altay T Smithason S Moore SK Ransohoff RM. Depletion of Ly6G/C(+) cells ameliorates delayed cerebral vasospasm in subarachnoid hemorrhage. J Neuroimmunol. (2011) 232:94–100. 10.1016/j.jneuroim.2010.10.016 21059474 PMC3053416

[7] Kwon MS Woo SK Kurland DB Yoon SH Palmer AF Banerjee U. Methemoglobin is an endogenous toll-like receptor 4 ligand-relevance to subarachnoid hemorrhage. Int J Mol Sci. (2015) 16:5028–46. 10.3390/ijms16035028 25751721 PMC4394463

[8] Fujimoto M Shiba M Kawakita F Liu L Shimojo N Imanaka-Yoshida K. Effects of tenascin-c knockout on cerebral vasospasm after experimental subarachnoid hemorrhage in mice. Mol Neurobiol. (2018) 55:1951–8. 10.1007/s12035-017-0466-x 28244007

[9] Gu X Zheng C Zheng Q Chen S Li W Shang Z. Salvianolic acid A attenuates early brain injury after subarachnoid hemorrhage in rats by regulating ERK/P38/Nrf2 signaling. Am J Transl Res. (2017) 9:5643–52. 29312516 PMC5752914

[10] Shao A Wang Z Wu H Dong X Li Y Tu S. Enhancement of autophagy by histone deacetylase inhibitor trichostatin a ameliorates neuronal apoptosis after subarachnoid hemorrhage in rats. Mol Neurobiol. (2016) 53:18–27. 10.1007/s12035-014-8986-0 25399954

[11] Jing CH Wang L Liu PP Wu C Ruan D Chen G. Autophagy activation is associated with neuroprotection against apoptosis via a mitochondrial pathway in a rat model of subarachnoid hemorrhage. Neuroscience. (2012) 213:144–53. 10.1016/j.neuroscience.2012.03.055 22521819

[12] Carrillo-Vico A Calvo JR Abreu P Lardone PJ García-Mauriño S Reiter RJ. Evidence of melatonin synthesis by human lymphocytes and its physiological significance: possible role as intracrine, autocrine, and/or paracrine substance. FASEB J. (2004) 18:537–9. 10.1096/fj.03-0694fje 14715696

[13] Cheung RT. The utility of melatonin in reducing cerebral damage resulting from ischemia and reperfusion. J Pineal Res. (2003) 34:153–60. 10.1034/j.1600-079x.2003.00034.x 12614473

[14] Esposito E Paterniti I Mazzon E Bramanti P Cuzzocrea S. Melatonin reduces hyperalgesia associated with inflammation. J Pineal Res. (2010) 49:321–31. 10.1111/j.1600-079X.2010.00796.x 20666977

[15] Caballero B Vega-Naredo I Sierra V DeGonzalo-Calvo D Medrano-Campillo P Guerrero JM. Autophagy upregulation and loss of NF-kappaB in oxidative stress-related immunodeficient SAMP8 mice. Mech Ageing Dev. (2009) 130:722–30. 10.1016/j.mad.2009.09.001 19751754

[16] Yang S Tang W He Y Wen L Sun B Li S. Long non-coding RNA and microRNA-675/let-7a mediates the protective effect of melatonin against early brain injury after subarachnoid hemorrhage via targeting TP53 and neural growth factor. Cell Death Dis. (2018) 9:99. 10.1038/s41419-017-0155-8 29367587 PMC5833397

[17] Chen J Wang L Wu C Hu Q Gu C Yan F. Melatonin-enhanced autophagy protects against neural apoptosis via a mitochondrial pathway in early brain injury following a subarachnoid hemorrhage. J Pineal Res. (2014) 56:12–9. 10.1111/jpi.12086 24033352

[18] Geyik M Erkutlu I Geyik S Alptekin M Gezgin I Gok A. Paradoxical morphometric and antioxidative effects of melatonin on vasospasm in experimental subarachnoid hemorrhage. J Neurol Sci. (2015) 32:756–66.

[19] Macleod MR O'Collins T Howells DW Donnan GA. Pooling of animal experimental data reveals influence of study design and publication bias. Stroke. (2004) 35:1203–8. 10.1161/01.STR.0000125719.25853.20 15060322

[20] DerSimonian R Laird N. Meta-analysis in clinical trials revisited. Contemp Clin Trials. (2015) 45:139–45. 10.1016/j.cct.2015.09.002 26343745 PMC4639420

[21] Song F Gilbody S. Bias in meta-analysis detected by a simple, graphical test. Increase in studies of publication bias coincided with increasing use of meta-analysis. BMJ. (1998) 316:471. 9492690 PMC2665616

[22] Ayer RE Sugawara T Zhang JH. Effects of melatonin in early brain injury following subarachnoid hemorrhage. Acta Neurochir Suppl. (2008) 102:327–30. 10.1007/978-3-211-85578-2_62 19388339

[23] Ersahin M Toklu HZ Cetinel S Yüksel M Yegen BC Sener G. Melatonin reduces experimental subarachnoid hemorrhage-induced oxidative brain damage and neurological symptoms. J Pineal Res. (2009) 46:324–32. 10.1111/j.1600-079X.2009.00664.x 19215574

[24] Wang Z Ma C Meng CJ Zhu GQ Sun XB Huo L. Melatonin activates the Nrf2-ARE pathway when it protects against early brain injury in a subarachnoid hemorrhage model. J Pineal Res. (2012) 53:129–37. 10.1111/j.1600-079X.2012.00978.x 22304528

[25] Cai J He C Chen L Han T Huang S Huang Y. Melatonin mitigate cerebral vasospasm after experimental subarachnoid hemorrhage: a study of synchrotron radiation angiography. J Instrument. (2013) 8:C06004. 10.1088/1748-0221/8/06/C06004

[26] Chen J Chen G Li J Qian C Mo H Gu C. Melatonin attenuates inflammatory response-induced brain edema in early brain injury following a subarachnoid hemorrhage: a possible role for the regulation of pro-inflammatory cytokines. J Pineal Res. (2014) 57:340–7. 10.1111/jpi.12173 25187344

[27] Chen J Qian C Duan H Cao S Yu X Li J. Melatonin attenuates neurogenic pulmonary edema via the regulation of inflammation and apoptosis after subarachnoid hemorrhage in rats. J Pineal Res. (2015) 59:469–77. 10.1111/jpi.12278 26383078

[28] Dong Y Fan C Hu W Jiang S Ma Z Yan X. Melatonin attenuated early brain injury induced by subarachnoid hemorrhage via regulating NLRP3 inflammasome and apoptosis signaling. J Pineal Res. (2016) 60:253–62. 10.1111/jpi.12300 26639408

[29] Zhao L Liu H Yue L Zhang J Li X Wang B. Melatonin attenuates early brain injury via the melatonin receptor/Sirt1/NF-κB signaling pathway following subarachnoid hemorrhage in mice. Mol Neurobiol. (2017) 54:1612–21. 10.1007/s12035-016-9776-7 26867656

[30] Yu X. 俞 晓波,褪黑素在蛛网膜下腔出血早期脑损伤中抗坏死性凋亡和抗神经炎症的机制研究 (2017) 浙江大 学.

[31] Cao S Shrestha S Li J Yu X Chen J Yan F. Melatonin-mediated mitophagy protects against early brain injury after subarachnoid hemorrhage through inhibition of NLRP3 inflammasome activation. Sci Rep. (2017) 7:2417. 10.1038/s41598-017-02679-z 28546552 PMC5445068

[32] Shi L Liang F Zheng J Zhou K Chen S Yu J. Melatonin regulates apoptosis and autophagy via ROS-MST1 pathway in subarachnoid hemorrhage. Front Mol Neurosci. (2018) 11:93. 10.3389/fnmol.2018.00093 29632474 PMC5879134

[33] Macleod MR O'Collins T Horky LL Howells DW Donnan GA. Systematic review and meta-analysis of the efficacy of melatonin in experimental stroke. J Pineal Res. (2005) 38:35–41. 10.1111/j.1600-079X.2004.00172.x 15617535

[34] Barlow KM Esser MJ Veidt M Boyd R. Melatonin as a treatment after traumatic brain injury: a systematic review and meta-analysis of the pre-clinical and clinical literature. J Neurotrauma. (2019) 36:523–37. 10.1089/neu.2018.5752 29901413

[35] Ye ZN Wu LY Liu JP Chen Q Zhang XS Lu Y. Inhibition of leukotriene B4 synthesis protects against early brain injury possibly via reducing the neutrophil-generated inflammatory response and oxidative stress after subarachnoid hemorrhage in rats. Behav Brain Res. (2018) 339:19–27. 10.1016/j.bbr.2017.11.011 29133197

[36] Zhang ZY Sun BL Yang MF Li DW Fang J Zhang S. Carnosine attenuates early brain injury through its antioxidative and anti-apoptotic effects in a rat experimental subarachnoid hemorrhage model. Cell Mol Neurobiol. (2015) 35:147–57. 10.1007/s10571-014-0150-x 25179154 PMC11486197

[37] Guo ZN Jin H Sun H Zhao Y Liu J Ma H. Antioxidant melatonin: potential functions in improving cerebral autoregulation after subarachnoid hemorrhage. Front Physiol. (2018) 9:1146. 10.3389/fphys.2018.01146 30174621 PMC6108098

[38] Sena E Wheble P Sandercock P Macleod M. Systematic review and meta-analysis of the efficacy of tirilazad in experimental stroke. Stroke. (2007) 38:388–94. 10.1161/01.STR.0000254462.75851.22 17204689

[39] Lythgoe DJ Little RA O'shaughnessy CT Steward MC– Pharmacol Br J. Effect of U-74006F on oedema and infarct volumes following permanent occlusion of the middle cerebral artery in the rat. Br J Pharmacol. (1990) 100:454.

[40] Cui HJ He HY Yang AL Zhou HJ Wang C Luo JK. Efficacy of deferoxamine in animal models of intracerebral hemorrhage: a systematic review and stratified meta-analysis. PLoS ONE. (2015) 10:e0127256. 10.1371/journal.pone.0127256 26000830 PMC4441464

[41] Kamp MA Lieshout JHV Dibué-Adjei M Weber JK Schneider T Restin T. A systematic and meta-analysis of mortality in experimental mouse models analyzing delayed cerebral ischemia after subarachnoid hemorrhage. Transl Stroke Res. (2017) 8:206–19. 10.1007/s12975-016-0513-3 28138916

[42] Marbacher S. Animal models for the study of subarachnoid hemorrhage: are we moving towards increased standardization? Transl Stroke Res. (2016) 7:1–2. 10.1007/s12975-015-0442-6 26754973

[43] Sharma HS Muresanu DF Nozari A Castellani RJ Dey PK Wiklund L. Anesthetics influence concussive head injury induced blood-brain barrier breakdown, brain edema formation, cerebral blood flow, serotonin levels, brain pathology and functional outcome. Int Rev Neurobiol. (2019) 146:45–81. 10.1016/bs.irn.2019.06.006 31349932

[44] Marchesini V Disma N. Anaesthetic neuroprotection in children: does it exist or is it all just bad? Curr Opin Anaesthesiol. (2019) 32:363–9. 10.1097/ACO.0000000000000723 30893117

[45] Jiang M Sun L Feng DX Yu ZQ Gao R Sun YZ. Neuroprotection provided by isoflurane pre-conditioning and post-conditioning. Med Gas Res. (2017) 7:48–55. 10.4103/2045-9912.202910 28480032 PMC5402347

[46] Olsen RW Li GD. GABA(A) receptors as molecular targets of general anesthetics: identification of binding sites provides clues to allosteric modulation. Can J Anaesth. (2011) 58:206–15. 10.1007/s12630-010-9429-7 21194017 PMC3033524

[47] Muir WW. NMDA receptor antagonists and pain: ketamine. Vet Clin North Am Equine Pract. (2010) 26:565–78. 10.1016/j.cveq.2010.07.009 21056300

[48] Yeleswaram K McLaughlin LG Knipe JO Schabdach D. Pharmacokinetics and oral bioavailability of exogenous melatonin in preclinical animal models and clinical implications. J Pineal Res. (1997) 22:45–51. 10.1111/j.1600-079X.1997.tb00302.x 9062870

