# Supplementary figures and images for: Efficacy of Melatonin in Animal Models of Subarachnoid Hemorrhage: A Systematic Review and Stratified Meta-Analysis
Source: Front Neurol. 2021 Sep 3;12:685731. doi: 10.3389/fneur.2021.685731 (PMC8446273; doi:10.3389/fneur.2021.685731)

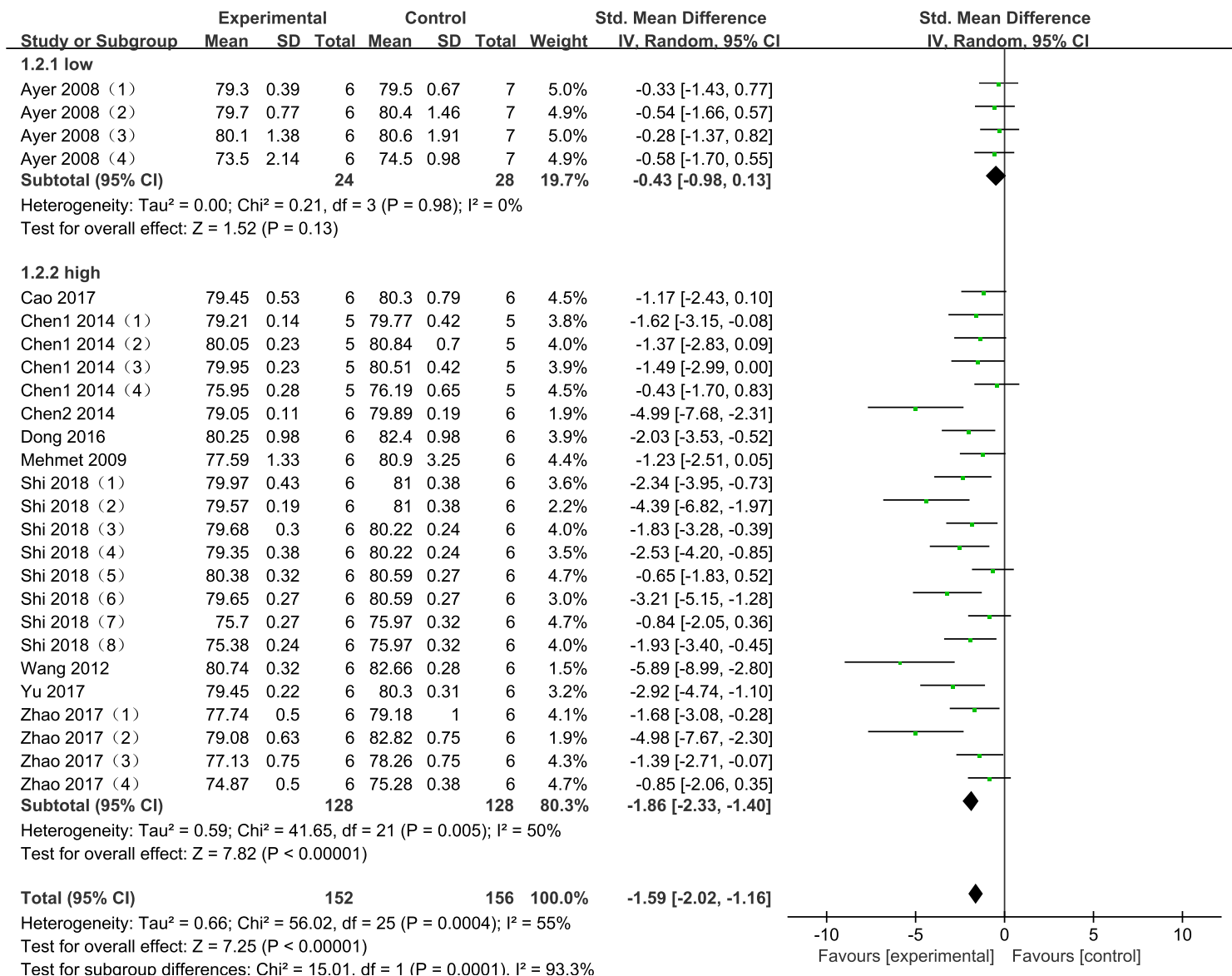

Supplement: Supplementary file 1 [file Data_Sheet_1.PDF]

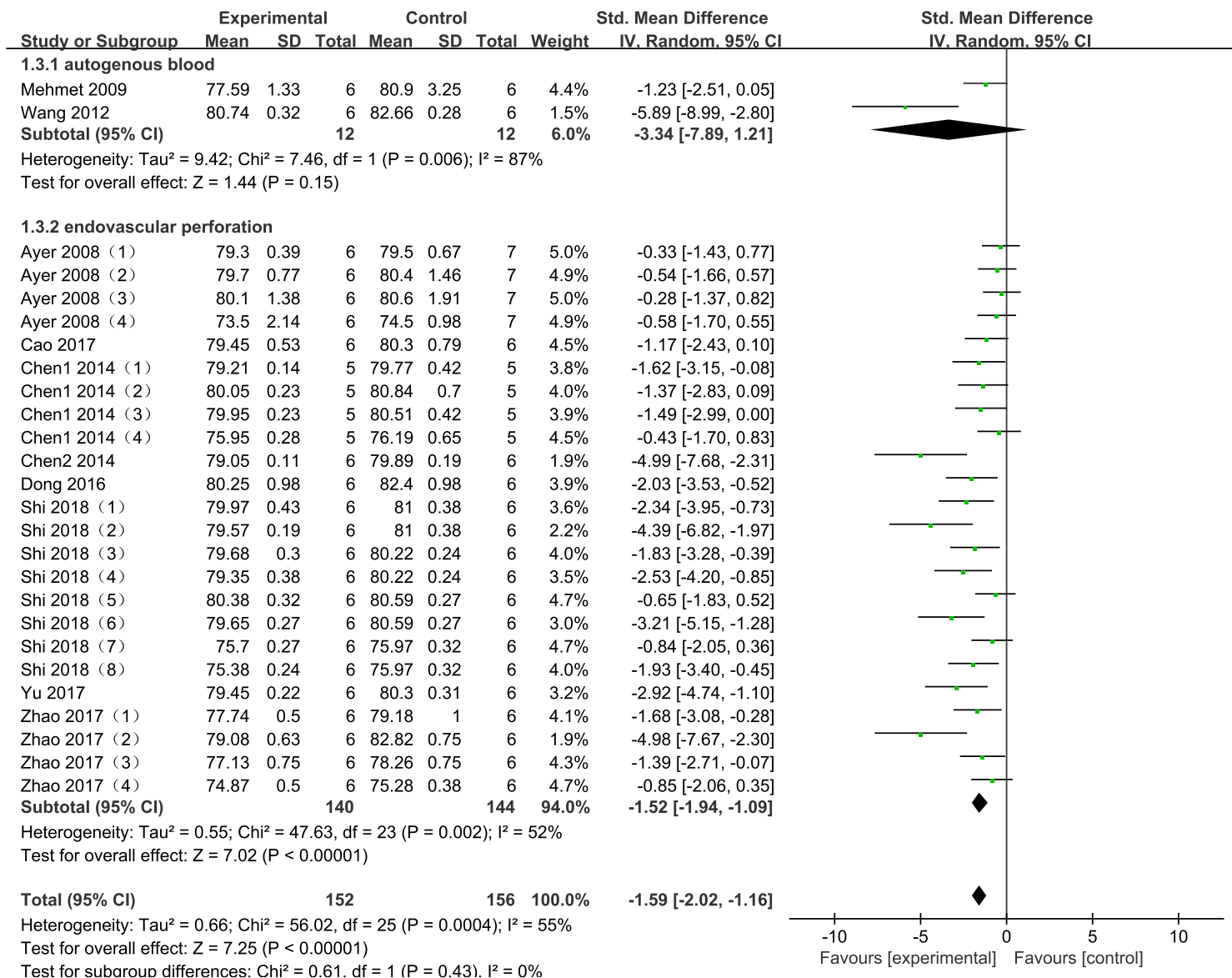

Supplement: Supplementary file 2 [file Data_Sheet_2.PDF]

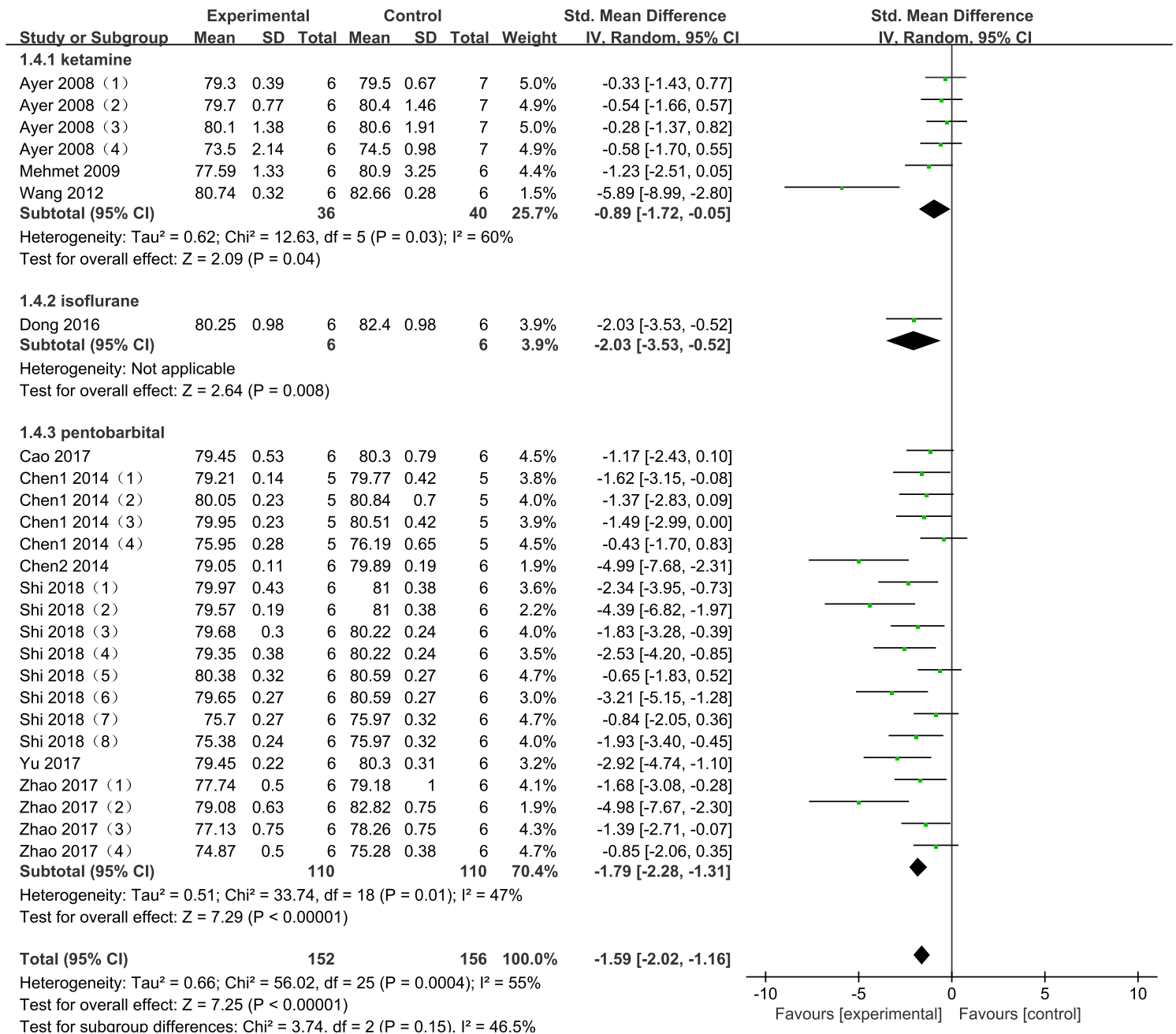

Supplement: Supplementary file 3 [file Data_Sheet_3.PDF]

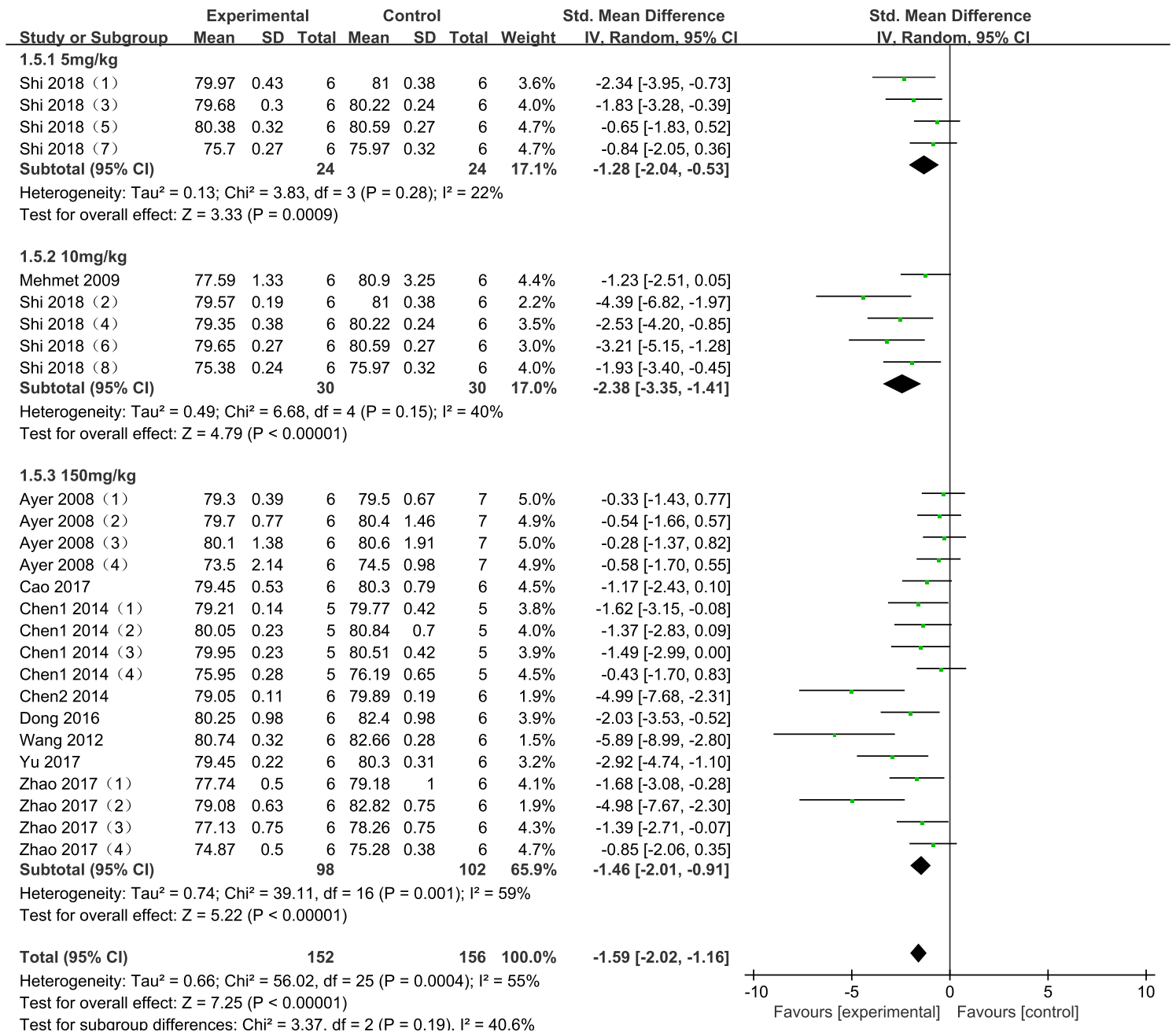

Supplement: Supplementary file 4 [file Data_Sheet_4.PDF]

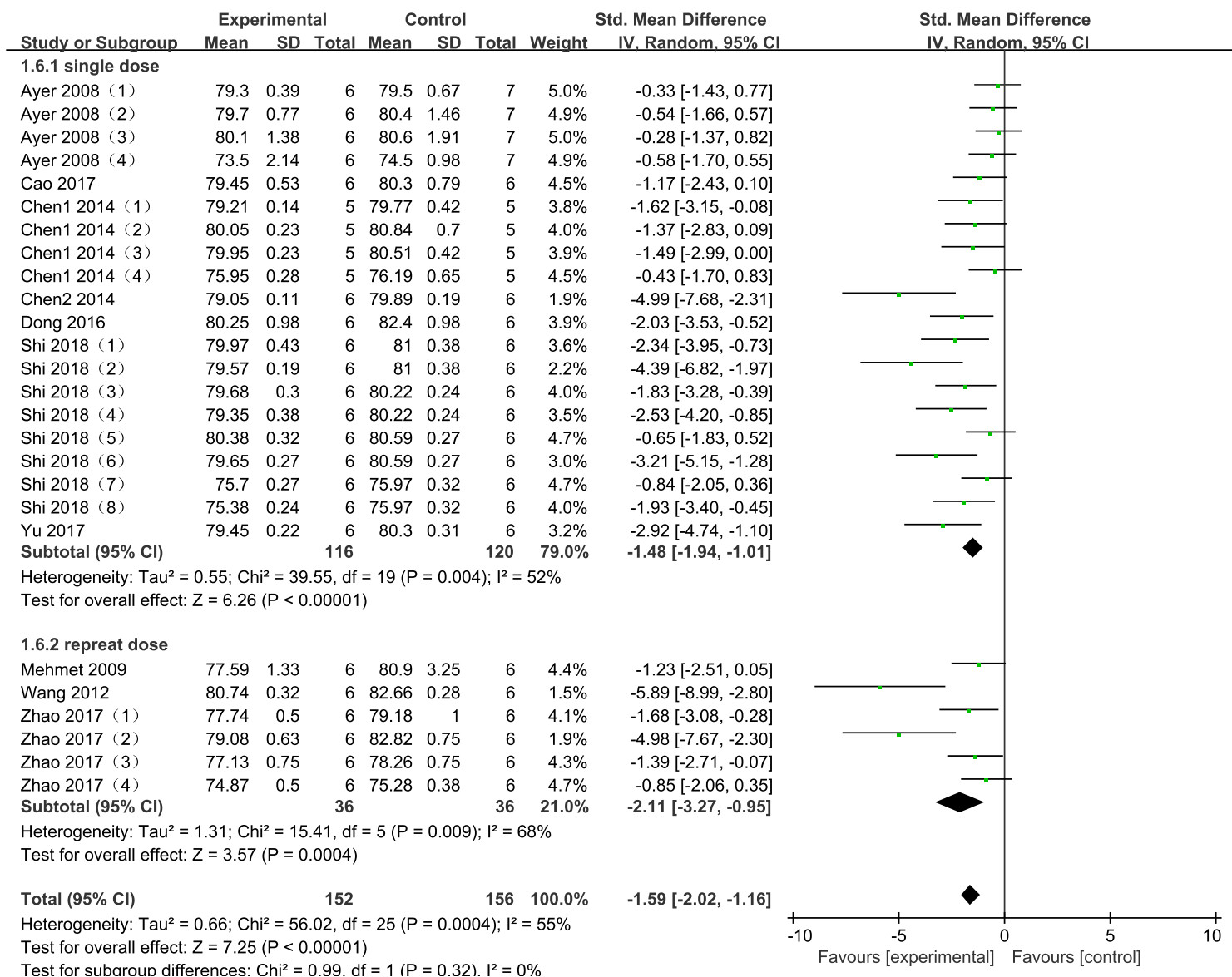

Supplement: Supplementary file 5 [file Data_Sheet_5.PDF]

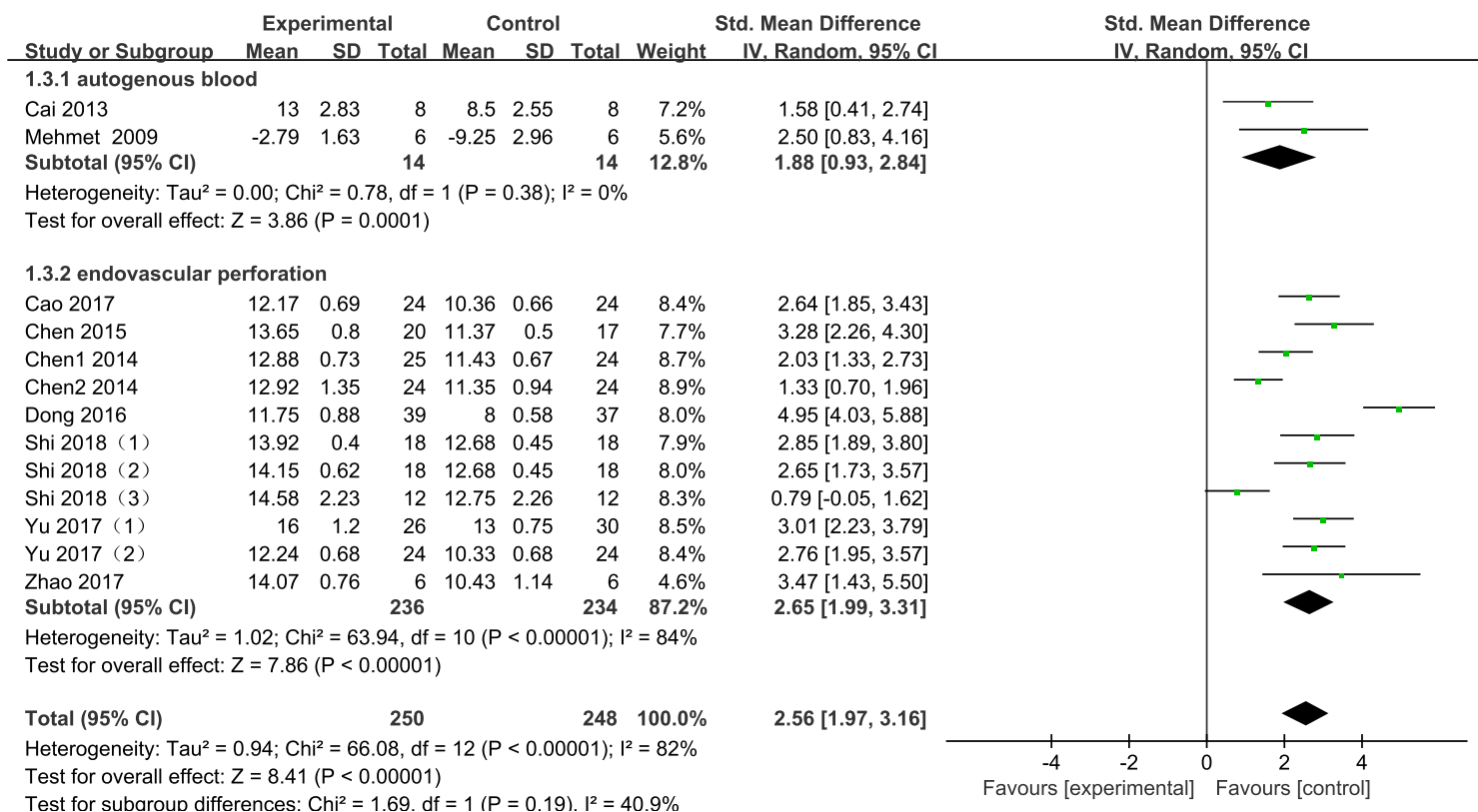

Supplement: Supplementary file 6 [file Data_Sheet_6.PDF]

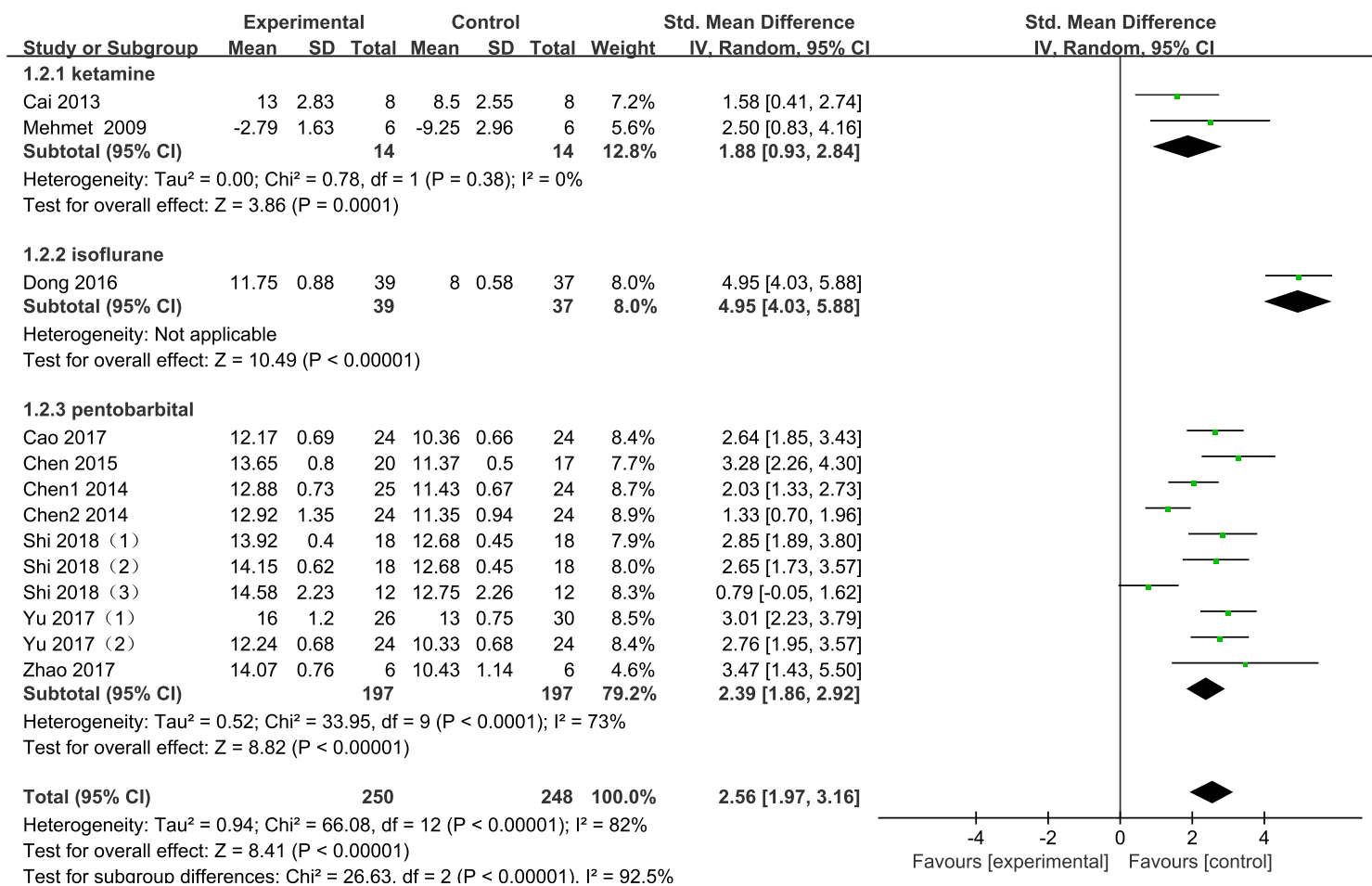

Supplement: Supplementary file 7 [file Data_Sheet_7.PDF]

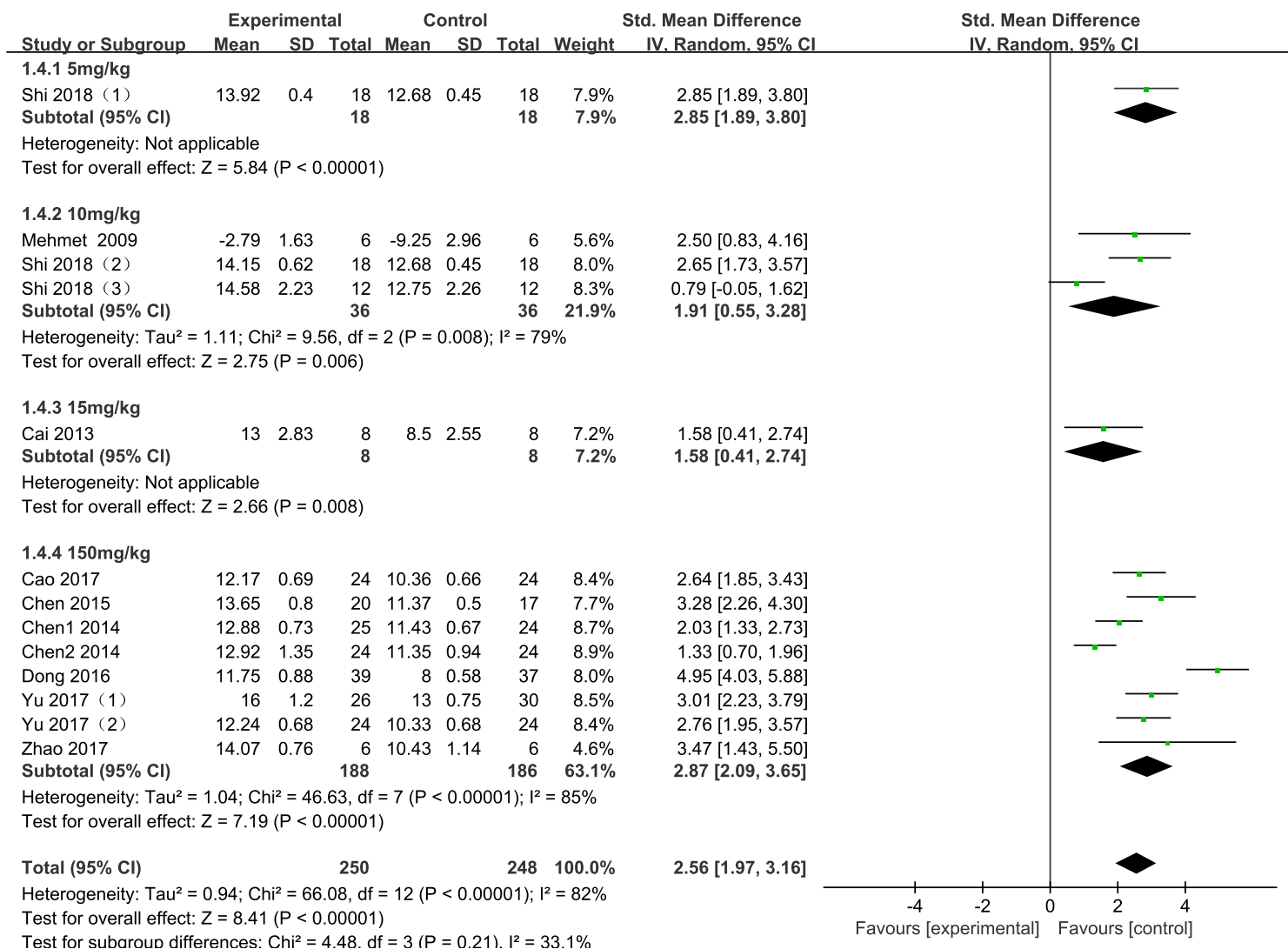

Supplement: Supplementary file 8 [file Data_Sheet_8.PDF]

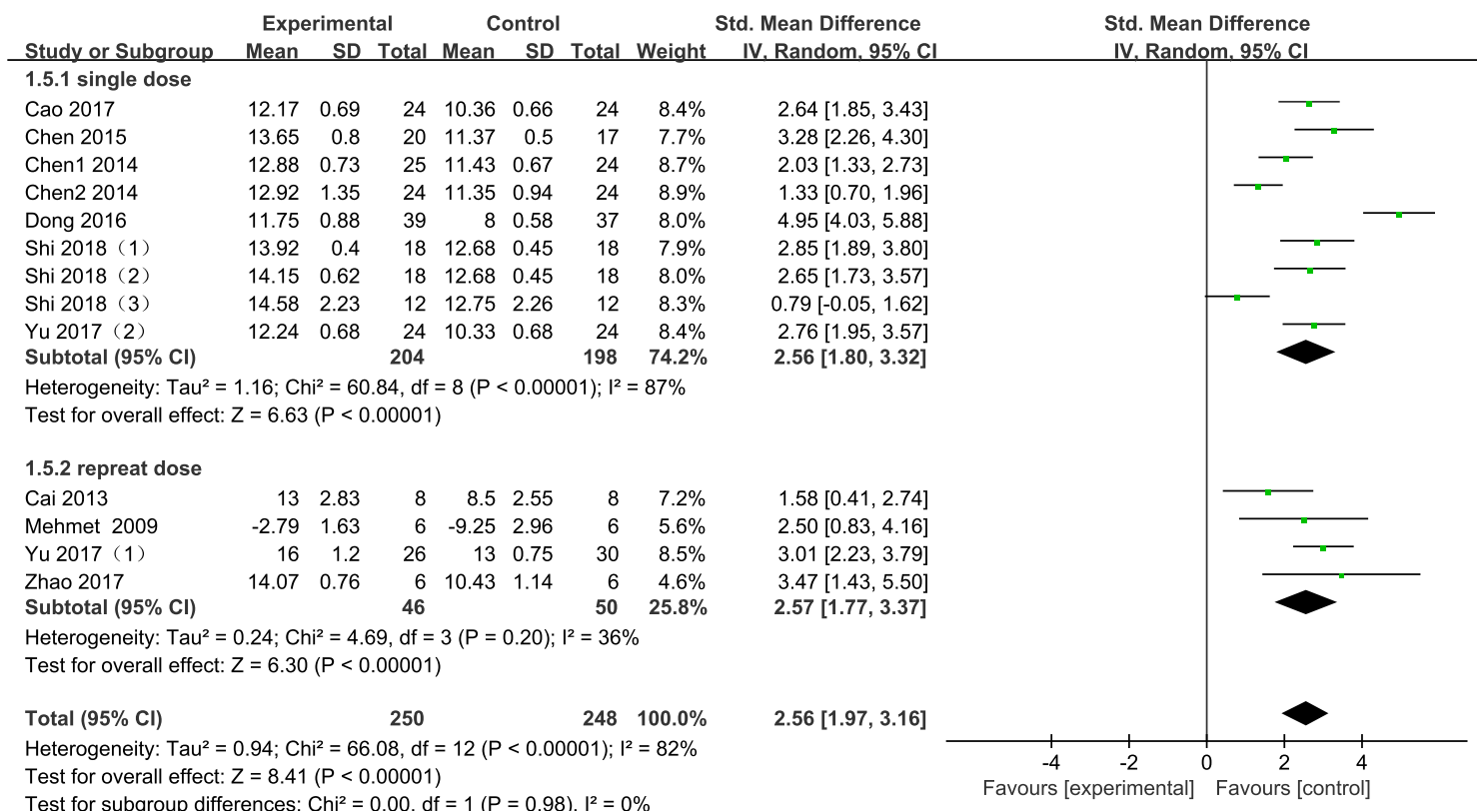

Supplement: Supplementary file 9 [file Data_Sheet_9.PDF]
