# Supplementary material for: Efficacy of Melatonin in Animal Models of Subarachnoid Hemorrhage: A Systematic Review and Stratified Meta-Analysis
Source: Front Neurol. 2021 Sep 3;12:685731. doi: 10.3389/fneur.2021.685731 (PMC8446273; doi:10.3389/fneur.2021.685731)
Supplement: Supplementary file 10 [file Data_Sheet_1.doc]

Pubmed:

(subarachnoid h(a)emorrhage[Title/Abstract] OR SAH[Title/Abstract] OR aneurysm[Title/Abstract] OR subarachnoid hemorrhage[MeSH Major Topic]) AND (melatonin[Title/Abstract] OR N-acetyl-5-methoxytryptamine[Title/Abstract] OR melatonergic agent[Title/Abstract] OR melatonin receptor agonist[Title/Abstract] OR melatonin[MeSH Major Topic]) NOT human[Title/Abstract] NOT patient[Title/Abstract] = 36

Web of Science:

#1 TS= (subarachnoid hemorrhage OR SAH OR subarachnoid haemorrhage)

#2 TS= (melatonin OR N-acetyl-5-methoxytryptamine OR melatonergic agent OR melatonin receptor agonist NOT human NOT patient)

#3=#1 AND #2 = 73

Embase:

#1 'subarachnoid hemorrhage':ab,ti OR 'subarachnoid haemorrhage':ab,ti OR sah:ab,ti

#2 (melatonin:ab,ti OR 'melatonin receptor agonist':ab,ti OR 'n acetyl 5 methoxytryptamine':ab,ti OR 'melatonergic agent':ab,ti) NOT patient:ab,ti NOT human:ab,ti

#3=#1 AND #2 = 36

CNKI:

SU='蛛网膜下腔出血'*'褪黑素' = 15
